# Towards a decision aid for self‐tests: Users’ experiences in The Netherlands

**DOI:** 10.1111/hex.12903

**Published:** 2019-06-13

**Authors:** Willemijn M. den Oudendammer, Jacqueline E. W. Broerse

**Affiliations:** ^1^ Faculty of Science, Athena Institute Vrije Universiteit Amsterdam Amsterdam The Netherlands

**Keywords:** consumer experience, consumer information, decision making, diagnostic test, qualitative research, self‐management, Self‐test

## Abstract

**Background:**

Self‐tests enable the identification of (risk factors for) diseases and are carried out on the user's initiative without medical indication or advice and often unaided by a health professional. They are frequently used, and their availability and usage are expected to grow. Self‐testing has both advantages and disadvantages. Making a well‐informed decision about whether to self‐test and which self‐test to use is of major importance.

**Objective:**

To provide insights into the experiences of self‐test users, identifying reasons to self‐test and perceived (dis)advantages of using self‐tests and the information highlighted as relevant by self‐test users to make well‐informed decisions.

**Methods:**

In a qualitative study, 28 users of a wide variety of self‐tests shared their experiences in focus groups and interviews.

**Results:**

Perceived disadvantages of self‐testing included the following: a wide range of available self‐tests, lack of insights into their reliability and content, possibility of mistakes in administering them, possibility of false‐positive and false‐negative results, lack of clarity about how to interpret results and consequently what action to take and fear of not being taken seriously by a general practitioner. Self‐test aspects that were viewed as most important include informed decision making, user‐friendliness, usefulness and reliability of results.

**Conclusion:**

A decision aid for future self‐test users can help people make a deliberate decision on whether to use a self‐test and which particular self‐test to use from the wide range available. The government, health professionals, patient organizations, consumer organizations and citizens all have a role to play in the development and implementation of a decision aid.

## 1 INTRODUCTION

Self‐tests enable the identification of risk factors or early stages of diseases and are carried out on the user's initiative, without a medical indication or advice and often unaided by a health professional. There are many different types of self‐tests (see Table 1). Four types of self‐test on bodily material (eg blood, urine and saliva) can be distinguished: (a) over‐the‐counter tests, in which consumers are alone responsible for the execution and interpretation; (b) home‐collect tests, which require consumers to ship a sample of bodily material to a laboratory for analysis, after which they receive the results of the test by post or Internet; (c) direct‐access tests, which require consumers to provide a sample while physically in a laboratory setting for analysis, after which they receive test results (eg by post or Internet); and (d) street‐corner tests in which consumers may opt (without a doctor's recommendation) to have their sample taken in a public place (eg supermarket or drug store) by trained personnel, after which they immediately receive the results.^1^ In addition, there are various types of self‐tests that do not involve bodily material. These include health‐related questionnaires which assess risk factors for certain conditions (eg cardiovascular disease), give insight into the overall health status of a person or assist in the diagnosis of conditions (eg mental health conditions), as well as various health checks offered by employers, gyms and private clinics (eg total body scans, measurement of body mass index and blood pressure). If costs are involved, self‐tests usually need to be paid out of pocket (not reimbursed by health insurance).^2^, ^3^

**Table 1:** Inclusion of self‐tests per category

Self‐test on bodily material			
Over‐the‐counter test: the consumer buys and conducts the self‐test, and interprets the results himself^a^	Home‐collect tests: the consumer takes bodily material himself, sends it to a laboratory where the test is conducted and results are interpreted	Direct‐access tests: the consumer lets the test be conducted and results interpreted at a laboratory	Street‐corner tests: the consumer lets the test be conducted and results interpreted at a public location
Health‐related questionnaires			
Questionnaires that assess risk factors for conditions	Questionnaires that give insight into the overall health status	Questionnaires that diagnose conditions	
Health check outside routine health care			
Overall health checks offered by the health insurer^b^	Overall health checks offered by the employer	Overall health checks offered by the gym	Overall health checks offered by a private clinic

a People using pregnancy tests or tests to monitor an existing disease were excluded.

b People using tests within the framework of national screening programmes offered by the government were excluded.

Over the past decade, self‐tests have become more readily available. First, there is an increase in both the number of conditions one can diagnose through self‐tests and the number of self‐tests on the market for each condition. In the UK, 104 self‐tests were identified in an Internet survey, which is related to 24 conditions.^4^ Second, locations that provide self‐tests are becoming more abundant: pharmacies and online stores increasingly sell self‐tests; street‐corner tests are increasingly offered; private clinics regularly advertise for total body scans; and the Internet is now full of websites which offer diagnostic/risk factor questionnaires. Moreover, the availability of self‐tests is expected to continue to grow.^5^, ^6^

In line with the growing availability of self‐tests is their frequent use. In a cross‐sectional survey in the Netherlands, 16% of the 7919 participants indicated having at some time used a self‐test on bodily material.^1^ The same study showed that 17% of those who had never used a self‐test expected to do so in the future, and 54% even stated a preference for self‐tests over other forms of testing. A questionnaire survey in the UK showed that 47 per 1000 women and 22 per 1000 men have at some point used a self‐test on bodily material (other than a pregnancy or high blood pressure test).^7^ The most frequently used self‐tests in Germany, the UK and the Netherlands include those for cholesterol, glucose, diabetes, kidney disease, HIV, chlamydia, lactose intolerance, allergies, vaginal infection and urinary infection.^1^, ^7^, ^8^, ^9^

The frequent use of self‐testing might be connected to an advantage that is often mentioned: convenient alternative for visiting a GP and taking responsibility for one's own health.^2^, ^7^, ^10^, ^11^ For example, after testing positive for risk factors, one might take responsibility and change one's health behaviour in order to avoid disease. The idea of taking responsibility for one's own health, often connected to the concepts of self‐management and autonomy in health, is in line with the position increasingly taken by governments in most welfare states (including the Netherlands), which encourage citizens to take responsibility for their own health and their health behaviour.^12^

There are, however, also a number of potential disadvantages to self‐testing. False‐positive results may cause distress in the self‐tester and lead to higher costs in the health system for unnecessary follow‐up tests.^8^ Delay in treatment may be caused by false‐negative results, either when the self‐test user is wrongly reassured, either because the test is inaccurately applied, or because the test results are incorrectly interpreted. A true‐negative result may also lead to delay in treatment when a self‐test is carried out for the wrong condition or if the results are misinterpreted.^6^, ^13^

Due to the increasing use of self‐tests and their rapidly growing availability, it is crucial to counteract the disadvantages of self‐testing; self‐test users should be enabled to make informed decisions about whether to self‐test and which self‐test to use. However, what information self‐test users need is not clear. As stated by Grispen et al, it is important that “consumers’ information use and needs concerning self‐testing should be identified, in order to provide a solid basis for informed‐choices on self‐testing.”^10^ Several other scholars have also pointed out the need of identifying relevant information to help future self‐test users make well‐informed decisions on self‐testing, for example by developing a decision aid.^6^, ^10^, ^11^, ^14^ Our study aims to acquire insights into the experiences of self‐test users, their main reasons to self‐test and perceived (dis)advantages of using self‐tests, as well as to identify information highlighted as relevant by self‐test users to make well‐informed decisions.

## 2 methods

Using a qualitative research design, semi‐structured interviews (SSIs) and focus groups (FGs) were conducted.

### 2.1 Inclusion

To gain a broader insight into experiences of a diverse group of self‐test users, maximum variation sampling was applied. Participants were invited if they met the following inclusion criteria:

- The self‐test belonged to one of the following categories: (a) self‐test on bodily material, (b) health‐related questionnaires and (c) health check outside routine health care (see Table 1).
- The self‐tests had been carried out in the last four years, except for the health‐related questionnaires, which should have been carried out within the last two years, since this type of self‐testing is more frequently used.

### 2.2 Recruitment

Participants were recruited through calls on the websites of two major Dutch patient organizations and one Dutch consumer association. Most of the participants had read the announcement on one of these websites and applied to be a study participant. Several participants were made aware of this study by acquaintances. Researchers checked each applicant on the inclusion criteria before including them as a study participant.

### 2.3 Data collection

FGs are an appropriate method for in‐depth exploration of perspectives of a relatively homogenous group of participants, as it encourages participants to share their perspectives openly and stimulates interactions and creativity. However, for self‐tests within the category “health check outside routine health care” and the “genetic tests” within the category of self‐test on bodily material, we conducted individual interviews since these tests are considered more sensitive and less culturally acceptable, which may constrain openness among participants.

The FGs and SSIs covered the same four themes: (a) most important reasons to decide to use a self‐test, (b) positive experiences and aspects of using a self‐test, (c) negative experiences and aspects of using a self‐test and (d) identification of most important aspects of self‐tests based on the previous steps. FGs and SSIs commenced with a short questionnaire that asked participants about their demographics and type of self‐test used. Throughout the FGs and SSIs, there was room for participants to bring up other aspects of self‐tests that arose. These were noted by the interviewer/facilitator to be added in the analysis when deemed appropriate. In total, 28 test‐users participated in this study in two FGs (n=13) and three SSIs in the category of self‐test on bodily material, two FGs (n=9) in the category of health‐related questionnaires, and three SSIs in the category of health checks outside routine healthcare (see Table 2).

**Table 2:** Specification self‐tests used by participants

**Self‐test on bodily material**	**FG1 ** ***n *** **=** *** *** **7** **FG2 ** ***n *** **=** *** *** **6** **SSIs ** ***n *** **=** *** *** **3**	**In the 2 FGs: seven women and six men, aged 21‐72** **In the SSIs: three women aged 28‐35**
‐ Over‐the‐counter‐test	*n *=* *13	Kidney function; Celiac disease; Cholesterol level; Proteins and bacteria in urine
‐ Home‐collect‐test	*n *=* *1	Blood in faeces
‐ Direct access test	*n *=* *7	Allergies; Glucose level; Rheumatism; BSE (bovine spongiform encephalopathy, mad cow disease); CRP level (C‐reactive protein, inflammatory level); Carrier test for cystic fibrosis
‐ Street‐corner test	*n *=* *5	Cholesterol level; Glucose level
**Health‐related questionnaires**	**FG3 ** ***n *** **=** *** *** **5** **FG4 ** ***n *** **=** *** *** **4**	**In the 2 FGs: Four women and five men, aged 42‐73**
‐ Risk assessment	*n *=* *5	Risk for diabetes; Risk for heart diseases
‐ Overall health status	*n *=* *8	Health status; Life style; Psychological status
‐ Diagnosis	*n = 6*	Depression; Proctology related conditions, Sleeping disorder
**Health check outside routine healthcare**	**SSIs ** ***n *** **=** *** *** **3**	**One woman and two men, aged 47–79**
‐ Overall health‐check offered by a private clinic	*n *=* *3	Total body scan; Health check for the ageing man, including: cholesterol, glucose level, BMI, stamina, PSA (prostate specific antigen)

As this study was deemed “non‐invasive” by Dutch law, and all participants were above 18 years of age, we did not require approval from a formal medical ethical committee.^14^ The researchers adhered to the national Code of Ethics for Research in the Social and Behavioural Sciences involving Human Participants.^15^ All participants received verbal and written information about the aim of the study in advance and the possibility to withdraw from the study at any time. Participants gave consent to audio tape the FG or interview and to use anonymized quotes. Within three weeks after every FG or interview, a summary was sent to the participant(s) for a member check; participants were free to make additions, remarks or changes. Comments on the summaries were included in further analysis.

### 2.4 Analysis

All FGs and SSIs were audiotaped and transcribed. Content analysis using thematic and open coding was used to analyse the transcripts with qualitative software MAXQDA. Two researchers read and re‐read all transcripts. A coding scheme was made by each researcher individually based on the first two transcripts, and after comparison and discussion, a unified coding scheme was developed. While coding the other transcripts, the coding scheme was regularly discussed and adjusted based on new findings.

## 3 results

### 3.1 Reasons to self‐test

The participants voiced various reasons for choosing a self‐test and revealed several advantages of self‐testing (see Table 3). The most frequently mentioned reasons were to diagnose or obtain reassurance of (not) having a health condition (ie disease or risk factor); the ability to gain insight into and knowledge of one's health status; possible prevention of a health condition and the ability to take action; and the convenience of self‐testing.

**Table 3:** Reasons to self‐test and perceived advantages of self‐testing per type of self‐test

Experience and explanation by participants	Self‐test on bodily material	Health‐related questionnaire	Health check outside routine health care
*Diagnosis/reassurance* Linking experienced symptoms to a health condition and using a self‐test to diagnose or reassure themselves and thus providing a sense of relief	X	X	X
*Gain insight* Even though no complaints are experienced, it is good to have insight into one's own health status	X	X	X
*Curiosity* Even though not actively looking for a self‐test and no complaints are experienced, curiosity may be triggered when self‐tests or offered	X	X	
*Prevention/action* Being able to take action and possibly prevent health conditions (eg by lifestyle changes) based on self‐test results gives a strong positive feeling (doing something right). Motivation to take action is higher after self‐testing than after testing in regular health care	X	X	X
*Autonomy and independence* Taking responsibility for one's own health and a feeling of independence and freedom (eg to choose whether or not to test and which test). Autonomy over one's own body and being the professional over on one's body. Important reason to *not* chose for testing in regular health care	X		X
*Convenience* Shorter timeline from deciding to want to test until obtaining results. Being able to test where and when it is convenient (eg in the comfort and privacy of one's own home)	X	X	X
*No GP* Having an uneasy feeling or being scared to visit a GP. Not wanting to bother a GP. GPs do not offer many preventive tests, especially not without specific symptoms	X		X
*Costs* Costs of self‐testing might be lower than when testing in regular health care due to a doctor's consult and laboratory analysis. At the same time, out‐of‐pocket payment for self‐testing is not considered as negative	X		

### 3.2 Perceived disadvantages

The disadvantages experienced by the participants are divided into four stages of self‐testing: (a) deciding to self‐test; (b) conducting the self‐test; (c) obtaining the self‐test results; and (d) after the self‐test. Aspects that did not fit into these four stages are mentioned separately.

#### 3.2.1 Deciding to self‐test

At this stage, people are deciding whether or not to use a self‐test and which one would be appropriate. In the categories of self‐tests on bodily material and health‐related questionnaires, the wide range of self‐tests on the market was often experienced as a disadvantage. For the first category, it was stated that many manufacturers produce self‐tests resulting in differences in costs as well as in the quality and reliability of the tests, into which the potential users had no insight.

What I also find difficult [in the wide range of self‐tests] is to separate the wheat from the chaff. (P5, FG1/2)

For the health‐related questionnaires, the lack of insight into the self‐tests’ reliability was for example caused by not knowing who has developed the questionnaire:

You have to keep on searching: from which website do they come [the questionnaires], because you often see that they are copies from other websites and then you hope to finally somewhere find the actual source. (P2, FG3/4)

In both categories of self‐tests, participants perceived that lack of clarity on the content of the test made it harder to choose which self‐test to use. It was mentioned that self‐test user manuals or the goal of a questionnaire were not clear.

For self‐tests on bodily material, it was mentioned that when a decision to do so was made, it was sometimes not possible to purchase self‐tests online from a Netherlands‐based supplier. For health checks outside the formal health service, a disadvantage concerned the lack of clarity on what information the user needs to provide in order to have the health check carried out.

#### 3.2.2 Administering the self‐test

At this stage, people are administering the self‐test themselves or are having it carried out at a laboratory or private clinic. Many users of self‐tests on bodily material and of health‐related questionnaires mentioned that an important disadvantage concerned the possibility of making mistakes in administering the self‐test. For the former category, this was due to unclear user manuals, especially when the test comprises several steps, which is often the case. Second, because most self‐tests are single‐use and might have been expensive, most users tend to be anxious about carrying it out correctly the first time which, on the contrary, makes them more prone to making mistakes. For health‐related questionnaires, the way the questions are posed and the answer options provided may lead to mistakes: the terms that are used may be open to interpretation and jargon, long sentences and double negatives are often used. Moreover, answer options can be subjective, such as “a lot versus a little,” or “often versus sometimes”.

For example with the pain I experienced, they go and ask: ‘How many pressure points do you experience?’ Pressure points… pressure points…? Yes, okay, I can sort of imagine what they mean, but how am I supposed to feel that? When I touch it hard or soft and in how big an area? Sort of understanding what they mean is not enough; you need to know exactly and that is not always the case. (P1, FG3/4)

Some level of discomfort was experienced when carrying out certain self‐tests on bodily material, for example when having to provide one's own blood sample.

For health‐related questionnaires, many participants said that they found it annoying when more time was required to fill out a test than stated beforehand. Furthermore, questionnaires could be perceived as untrustworthy and unreliable because of how the questions were posed—for example when questions came across as superficial, or were suggestive and the users felt that it became clear from the line of questioning or the answer options which answer would be “the best one”.

They ask for the sake of asking […] for example that stuff about eating healthy [eating two pieces of fruit a day]… You already know what they want to hear… (P5, FG3/4)

Questionnaires on mental health issues were considered unreliable by several participants because the answers strongly depended on the user's state of mind at the moment of filling in the questionnaire.

For health checks outside the formal health system, a participant mentioned that it was unclear exactly what test was carried out. It was stated that his blood would be examined, but precisely what it would be tested for was not specified.

#### 3.2.3 Obtaining self‐test results

This stage focuses on how results are obtained or presented, the information they provide and how the results are interpreted by users. The unreliability of test results is identified as a disadvantage by many users of self‐tests on both bodily material and health‐related questionnaires. Participants stated that the reliability of the results cannot always be assured due to mistakes in conducting the test‐steps carried out wrongly, or a question or answer option misunderstood. Moreover, users of health‐related questionnaires noticed that there are numerous questionnaires on many health issues. When filling out several of them, the results can differ, since the cut‐off for (not) having a health condition may differ.

The contradiction: one questionnaire states for example that to have fibromyalgia you need to have 18 of these points where it hurts. Than you go to another website and they say 15. Hello…what is it going to be…15…18…? One questionnaire raised questions that the other one didn’t. Yes well, you get insecure about that: which one is right? (P1, FG3/4)

As a result, some participants worried about false‐positive or false‐negative results. Becoming unnecessarily worried due to a false‐positive result was mentioned as an important disadvantage. None of the participants experienced a wrong result themselves, as far as they knew. At the same time, they wondered how they can be sure since they did not always consult a GP after a self‐test.

It may also be unclear what test results mean. Some users of self‐tests on bodily material and health‐related questionnaires found that instructions on how to interpret the results and advice on what action should or could be taken on the basis of the results were lacking. Moreover, users of questionnaires regularly mentioned that the results very often stated that a GP should be consulted to verify the results if the user was doubtful or worried about them, or when the results were not what they expected. It made users wonder about the benefit of taking a self‐test if it is almost always advised to consult a GP.

There are two important disadvantages related to health‐related questionnaires. First concerns were raised about the possible hidden commercial interest. Some participants felt that the result of a questionnaire pushed them in the direction of a certain product. Second, the participants mentioned the issue of privacy. Users often felt that not enough information was provided on what was being done with their data. Since most questionnaires are filled out online, some participants felt that the data may be stored somewhere and may serve another purpose of which they are unaware and for which they had not given permission.

Two users of health checks outside the formal health system were dissatisfied with how the test results were communicated. One participant mentioned that some results in which he had no interest were communicated and the possible action based on the results was discussed, while he had no wish to hear about this.

After the diagnosis based on the results he [the doctor] came with a whole story on what I should do. I didn’t ask for that, but he immediately started discussing it. So within 10 minutes it became clear that if I wouldn’t do anything, my life expectancy would go down by 6 years. I really was annoyed by this! (SSI2, P2)

Another participant using health checks outside the formal health system was negative about how a positive test result was communicated. He felt that the doctor was building up the tension regarding the results, which he experienced as very inappropriate.

#### 3.2.4 After the self‐test

This stage entails all aspects of self‐testing that play a role after the results are obtained. First, users of self‐tests on bodily material often mentioned the experience of stress without the support of a health professional as a disadvantage. Several participants explained that when the results are false‐positive, people will look up information about the condition which will lead to unnecessary worries. More importantly, they stated that whether or not results are false‐ or true‐positive, they would have to “carry” this knowledge “alone”:

I wrote down: ‘alone’. Because you are alone with it [the test results]. The ideal situation would be that you are on the same wavelength with your GP, so you can discuss and exchange thoughts and feelings. So when you are doing this test on your own, that already means…well mistrust is a big word, but you are not on the same page as your GP. So you have to deal with everything alone, interpret everything alone and then you may find yourself in a downwards spiral. And that, actually, is far from ideal. (P4, FG1/2)

Second, deciding what action to take based on the results was seen as a disadvantage by many participants. Consulting a GP based on the results was seen as a possibility, but not easy. Many users of self‐tests feared that their GP would not take them seriously or that their trust relationship with their GP would suffer, because some GPs might not appreciate people self‐testing.

I would see it as an issue of trust. I wouldn’t say cheating, but it comes rather close. (SSI2, P3)

Several users of self‐tests on bodily material and health checks outside the formal health system actually experienced their results not being taken seriously and the relationship with their GP becoming more difficult as a result.

I went to a GP with my results and he said: ‘oh you read this and you tested that…well we keep record of these things ourselves.’ No discussion was possible. (P2, FG1/2)

#### 3.2.5 Additional disadvantages

Many users of health checks outside the formal health system experienced the feeling of shame as a disadvantage of self‐testing throughout the whole process. This feeling was experienced towards their social surroundings, because self‐testing outside the formal health system is seen as out of the ordinary. For example, people addressed the personal costs involved and the commercialization of the tests. One participant experienced this especially when no condition was found in self‐testing, but later on was diagnosed within the formal health service.

And then of course I was taunted: ‘I told you so, did a total body scan…total waste of money.’ And I can imagine the doctor [within regular healthcare] also smirking: ‘you see, money spent, nothing was found and now we do find something is wrong.’ So you would not dare to tell anyone, would you? At least I wouldn’t. (SSI2, P3)

### 3.3 Important aspects of self‐tests

Aspects of self‐testing experienced as most important are divided into three main categories: informed decision making, user‐friendliness and reliability of results (see Table 4).

**Table 4:** Important aspects of self‐tests

Informed decision making
Information on content and results of self‐test
Independent comparison between self‐tests/clinics and comparison with regular health care
Information on usage of results
User‐friendliness
Clear instructions and format of self‐test
Easy and user‐friendly execution of self‐test
Usefulness and reliability of results
Specified results, including (possible) actions to be taken
Scientifically reliable results

#### 3.3.1 Informed decision making

Informed decision making was mentioned as an important aspect for all categories of self‐tests. Participants reflected on what information was needed in order to enable future self‐test users to make a better‐informed decision, and mentioned their ideas for how to improve self‐tests.

One of the aspects most often mentioned was the clarity of information on the content and the results of the self‐test. For self‐tests on bodily material and the health‐related questionnaires, it was emphasized that the purpose of the self‐test should be very clearly stated: what will the self‐test measure, what will the main topic of the questionnaire be and what will the results entail. Similarly, for health checks outside the formal health system, the need for clear and open communication regarding the different test options was underlined. In this way, the chosen self‐test will better fit the needs of the future user and it will be less likely that the results will give a false sense of security or lead to unnecessary worries. For the health‐related questionnaires, it was also mentioned that the source of the questionnaire should be clearly stated and users should be informed about how much time it will consume.

Second, it was perceived important to be able to compare the wide range of options. Quality assurance in the form of a certification mark by an independent institute was broadly considered desirable for self‐tests. Users of health checks outside the formal health system specifically stated that it is important for future users to be aware of the competition between clinics and the information on clinics’ websites should not be the main source of information. Rather, there should be a way to compare clinics, for example a comparison between the type of tests offered and possible health conditions to test for. Another comparison that participants recommended was between using a self‐test on bodily material versus being tested within the health system. In addition, for self‐tests on bodily material it should be clearly stated, especially when bought on the Internet, if the test is suitable for self‐use or is for professional use only.

The (possible) use of results is a third important aspect that needs to be clear in advance in order to make an informed decision. Users of self‐tests on bodily material as well as users of health checks outside the formal health system often mentioned the importance of being able to use their self‐test results in the formal health system and being taken seriously by health professionals.

Call the GP’s office to say: ‘I have done this or that self‐test, I expected these results, and these were the actual results. Do you feel that I should come by for an appointment or not?’ (P6, FG1/2)

For the health checks outside the formal system, it was specifically mentioned that it would be beneficial for the private clinic to provide information on the tests conducted and results to the user's GP when this is desirable.

With respect to health‐related questionnaires, users emphasized that it is important to make clear in advance if and how the results will be used by the provider of the questionnaire: specifically, whether the results be used for scientific purposes and (how) anonymity will be safeguarded. This was mentioned especially for online questionnaires, since it often is unclear what happens with information provided online.

#### 3.3.2 User‐friendliness

User‐friendliness was seen as an important aspect for self‐tests on bodily material and for health‐related questionnaires. First, the instructions of self‐tests should be clear. For self‐tests on bodily material, it is vital that the instruction manual provides step‐by‐step information and preferably uses pictures, to make it as easy to understand as possible. Health‐related questionnaires should follow a clear format and make clear which topics will be addressed. In addition, the design should be uncluttered, attractive and have a calm and easy‐to‐read font. Second, administering self‐tests should be easy and user‐friendly. The steps of carrying out the self‐test on bodily material should be simple and give as little discomfort as possible. The questions and answer options in the health‐related questionnaires should be understandable and unambiguous.

#### 3.3.3 Usefulness and reliability of results

The usefulness and reliability of the results were specifically mentioned as an important aspect by users of self‐tests on bodily material and health‐related questionnaires. For both categories, it was stated that the results should give clear information in order to be useful. Results which provide only a number, percentage or likelihood are not desirable; they should be specified in words, thus making sure the user understands what the results entail. Moreover, participants stated that it is of the utmost importance that results include which action(s) the user could or should take based on the results; otherwise, the results were seen as quite useless. Second, for self‐tests on bodily material it was mentioned that future users should make sure that the self‐test is scientifically reliable.

## 4 discussion

Participants were clearly aware of their own health, and they took responsibility for it and saw self‐management of their health as being important. This is in line with the literature, which shows a trend of people taking responsibility for and managing their own health.^2^, ^9^, ^10^, ^11^, ^12^ Whether or not people take action after a positive self‐test result often remains unclear in the literature. However, many of our study participants saw self‐test results as a cue to action; they continued their responsible health behaviour and often took immediate action after a positive test result, ranging from changing their lifestyle to consulting a GP.

The types of self‐tests included in this study differ from each other. The health‐related questionnaire stands out, since no physical health check is carried out. This type of self‐test may, however, be the one most often used by the general public, since barriers to use it are very low (it is free, can be carried out at home and is non‐invasive). Despite the differences, results regarding reasons to self‐test and disadvantages of self‐testing are quite similar between participants of different types of self‐tests.

Many reasons to self‐test identified in this study are also found in other studies,^9^ for example being uncertain about one's health and looking for reassurance or confirmation, not wanting to visit a GP, convenience and costs of self‐testing. Noteworthy is that in our study prevention and the ability to take action were of major importance, while in a German study^16^ these were less frequently mentioned reasons.

The participants in our study indicated several problematic issues during the four stages of self‐testing. These were experienced not only as obstacles for a good experience with self‐testing for themselves, but also as on‐going problems that future self‐test users would face when deciding whether to self‐test. Two issues that were raised when discussing disadvantages of self‐testing were not translated into important aspects of self‐tests that possible future self‐test users should bear in mind. The first is the experience of being alone with the test results. Several participants explained that when a result was positive, there is no one to provide (emotional) support. Second, there is a stigma experienced about using self‐tests. Users of all self‐test categories were afraid it would surface when they consulted a GP based on their results and some actually experienced this. As a consequence, they were reluctant to consult a GP based on a positive result. Concerning the health checks outside the formal system, the stigma regarding self‐testing was not only experienced within the formal health system, but also in the personal environment of the self‐test user. Friends or family members argued that the self‐test was unreliable or too expensive. The danger of stigma is the possible delay in treatment, when people are reluctant to consult a GP based on their positive self‐test result. Even though a decision aid cannot remove this burden, or help in coping with positive results, it is important to look into this issue in further research.

### 4.1 Well‐informed decision making

Although taking responsibility for one's own health is a trend which addresses the needs of the public and is encouraged by both health professionals and the Dutch government, support for citizens in well‐informed decision making concerning self‐testing seems to leave much to be desired. The Dutch health system responds rather ambivalently to self‐testing. Policymakers and health professionals are generally positive about people taking responsibility for their own health and GP consultation for preventive purposes is becoming more common, but in practice, the Dutch health system does little to facilitate and support the use of self‐tests. There are limited options for citizens to take a preventive test in the formal health system especially without an indication, and the Dutch government has not actively facilitated informed decision with regard to self‐testing.

Self‐testing is one of the ways for people to manage their own health. To achieve this, self‐testing should be more integrated in society and the health system, meaning that help and support are offered to (future) self‐testers to make a well‐informed decision on whether or not to self‐test and subsequently which self‐test to use. We found that the source of information on self‐tests mainly came from commercial and for‐profit organizations, such as self‐test manufacturers and organizations that offer self‐tests. We have shown that this leaves much to be desired according to self‐test users. We agree with Grispen et al^8^, ^13^ and Ickenroth et al^7^ that an overview of information on self‐tests and quality criteria should be made available from an independent source. As we have shown, self‐test users have a clear opinion on what is important when making a well‐informed decision. Therefore, we argue that their experiences should also be integrated in a practical tool for (future) self‐test users to make a well‐informed decision, such as a decision aid for future self‐test users. For relevant elements of such a decision aid, based on the perspective of self‐test users, we refer to the Appendix S1. Based on the results of this study, the involved patient organizations have taken up this challenge and have developed a website (www.checkdecheck.nl) that provides such a decision aid.

Governments and health care organizations also need to take responsibility in facilitating people's wish to manage their own health. An important role for the government is to set legal boundaries. First, awarding an independent quality or certification mark to self‐tests is something the government could do. This should be based on, among others, criteria provided by self‐test users, such as the important aspects of self‐tests shown in Table 4. In this way, comparisons between the broad range of self‐tests on bodily material, private clinics providing checks and different health‐related questionnaires do not have to be made by the future users themselves, but would be supported by government policy. Second, the government should ensure that certain information about self‐tests is made available (see Appendix S1). It is also important that in formal health settings, such as at the GP's surgery, the decision aid should be brought to the attention of possible future self‐test users.

### 4.2 Methodological considerations

In this study, triangulation of sources was achieved by including a wide diversity of test users (different backgrounds and different self‐tests) providing insights from various perspectives. For the category of self‐tests on bodily material and health‐related questionnaires, FGs were complemented with interviews for self‐tests on bodily material, providing triangulation of methods. Data saturation was achieved since no new aspects were brought up during SSIs and FGs. However, regarding the health checks outside formal care three interviews were carried out and it is unclear whether saturation was achieved. This relatively low number of participants was due to the limited response to the (repeated) calls for participants. This could be due to the low number of people in The Netherlands carrying out health checks outside the formal health system, but may also be linked to the stigma surrounding this category of self‐testing and the feelings of shame experienced by self‐test users in this category, as explained in the results under “additional disadvantages”.

## 5 conclusion

Self‐test use is frequently practised and is likely to increase in the coming years. Self‐testing is a way for people to take responsibility for their own health. Self‐testing is not in itself a positive or negative phenomenon and may have different implications in each individual case. In order for future self‐test users to make well‐informed decisions, the availability of a practical tool is highly relevant. Such a tool should include an overview of information on self‐tests and quality criteria, and also integrate the experiences of self‐test users. A decision aid may help to make a deliberate decision on whether to use a self‐test and which particular self‐test to use. The government, health professionals, patient organizations, consumer organizations and the general public all have a role to play in supporting informed decision making on self‐test. We believe that this study provides important first insights into the content of a decision aid, based on the experiences and needs of self‐test users. We encourage more in‐depth research into the impact of the use of such a decision aid.

## CONFLICT OF INTEREST

The authors declare that there is no conflict of interest.
