# Supplementary material for: Towards a decision aid for self‐tests: Users’ experiences in The Netherlands
Source: Health Expect. 2019 Jun 13;22(5):983–92. doi: 10.1111/hex.12903 (PMC6803405; doi:10.1111/hex.12903)
Supplement: Supplementary file 1 [file HEX-22-983-s001.docx]

**Annex. Towards a Decision Aid for self-tests**

The aspects of self-tests that are perceived as most important by the participants, can be used to develop a decision aid to help future self-test users to make a well-informed decision. In this annex the authors give first recommendations towards a decision-aid for possible future self-test users, based on experiences of self-test users and focussing on information that should be provided to the user.

First, we should differentiate between two stages of decision-making: (1) deciding to self-test or to turn directly to regular healthcare and (2) deciding between the categories of self-tests and deciding between the broad range of available self-tests. Therefore, the decision-aid should consist of two components.

The first component concerns the ability to make a well-informed and deliberate decision on whether to use a self-test. Several important aspects that have been mentioned by participants under ‘informed decision’ (as shown in table 4) can be used here. The decision aid should encourage possible users to ask themselves the following questions, and should provide ways to answer them:

1. Does the content of a self-test align with my needs;
2. Will the results of a self-test provide me with useful information;
3. Can I use the results in regular healthcare;
4. Are there other options, besides a self-test, that might fulfil my needs?

The second component should aid the decision regarding which self-test category would best fit the user’s needs. For all three categories of self-tests, participants mentioned specific questions that need to be answered, in order to decide whether or not this category would be the best fit for their need. If the answer to these questions is ‘no’, an alternative self-test category should be used. The decision aid should help provide answers to the following:

1. *Self-test on bodily material*: are there no other less invasive tests (i.e. not on bodily material) that will also fulfil my needs?
2. *Health-related questionnaires*: is a questionnaire available of which the source is a well-known institute and which is scientifically validated?
3. *Health checks outside routine healthcare*: comparison of clinics (exactly which tests are provided by which clinic) and do they fit my needs?

The second component furthermore aids in the decision in the broad range of self-tests within the chosen self-test category. It should be determined if self-tests comply with important aspects mentioned by participants under ‘user friendliness’ and ‘usefulness and reliability of results’ (as shown in table 4). There are two ways to determine if a self-test complies with these aspects: setting-up a checklist for future users, or to award a quality or certification mark to self-tests. The checklist ought to be included in the decision aid as a tool for future self-test users to decide by themselves whether a self-test should be used. At least information to answer the following questions should be available:

1. Are the instructions of a self-test on bodily material clear and is the format of the questionnaire clear;
2. Is the correct execution of the self-test easy and user friendly;
3. Will the results include specified information, including actions to be taken;
4. Is the reliability of the results scientifically guaranteed?

Awarding a quality or certification mark to self-tests should be based on, amongst other things, criteria shown in table 4 of the article and be taken up by the government.
